# Development of a knowledge-based healthcare-associated infections surveillance system in China

**DOI:** 10.1186/s12911-023-02297-y

**Published:** 2023-10-10

**Authors:** Yu Cao, Yaojun Niu, Xuetao Tian, DeZhong Peng, Li Lu, Haojun Zhang

**Affiliations:** 1 College of Computer Science, Sichuan University, No. 24 South Section 1, Yihuan Road, 610065 Chengdu, China; 2 LiLian Information Technology Company, Room 1536, Building 1, No.668 Shangda Road, Baoshan District, 201999 Shanghai, China; 3 The dean’s office, Second Provincial People’s Hospital of Gansu, No.1 Hezheng West Road, Chengguan District, 730099 Lanzhou, China; 4 Nosocomial Infection Management and Quality Control Center of Gansu Province, Lanzhou, China

**Keywords:** Healthcare-associated infections, Surveillance system, Knowledges rules, Infection clinical guidelines

## Abstract

**Background:**

In the modern era of antibiotics, healthcare-associated infections (HAIs) have emerged as a prominent and concerning health threat worldwide. Implementing an electronic surveillance system for healthcare-associated infections offers the potential to not only alleviate the manual workload of clinical physicians in surveillance and reporting but also enhance patient safety and the overall quality of medical care. Despite the widespread adoption of healthcare-associated infections surveillance systems in numerous hospitals across China, several challenges persist. These encompass incomplete coverage of all infection types in the surveillance, lack of clarity in the alerting results provided by the system, and discrepancies in sensitivity and specificity that fall short of practical expectations.

**Methods:**

We design and develop a knowledge-based healthcare-associated infections surveillance system (KBHAIS) with the primary goal of supporting clinicians in their surveillance of HAIs. The system operates by automatically extracting infection factors from both structured and unstructured electronic health data. Each patient visit is represented as a tuple list, which is then processed by the rule engine within KBHAIS. As a result, the system generates comprehensive warning results, encompassing infection site, infection diagnoses, infection time, and infection probability. These knowledge rules utilized by the rule engine are derived from infection-related clinical guidelines and the collective expertise of domain experts.

**Results:**

We develop and evaluate our KBHAIS on a dataset of 106,769 samples collected from 84,839 patients at Gansu Provincial Hospital in China. The experimental results reveal that the system achieves a sensitivity rate surpassing 0.83, offering compelling evidence of its effectiveness and reliability.

**Conclusions:**

Our healthcare-associated infections surveillance system demonstrates its effectiveness in promptly alerting patients to healthcare-associated infections. Consequently, our system holds the potential to considerably diminish the occurrence of delayed and missed reporting of such infections, thereby bolstering patient safety and elevating the overall quality of healthcare delivery.

**Supplementary Information:**

The online version contains supplementary material available at 10.1186/s12911-023-02297-y.

## Background

Healthcare-associated infections (HAIs) [1–4] are infections acquired during hospitalization or after discharge, but exclude those that have started before admission or are already incubated at access. HAIs are a significant public health issue worldwide, increasing morbidity, mortality, and healthcare costs. In recent years, there has been a growing interest in developing automated HAI surveillance systems to improve the detection and prevention of HAIs. These systems use standardized data collection and analysis methods to identify potential cases of HAIs, allowing healthcare providers to take prompt action to prevent further spread.

### Importance of HAIs surveillance

Hospital-acquired infections (HAIs) impose significant burdens on patients. For instance, in Argentina, bloodstream infections (BSI) lead to an average of 24.6% additional mortality and incur healthcare costs of $4888.42 for patients in intensive care departments [5]. In the United States, HAIs result in an estimated cumulative cost of nearly $10 billion per year [6].

Globally, the prevalence of HAIs varies, with estimates of 7% in developed countries and 10% in developing countries [3]. Specifically, the Netherlands reports an average prevalence of 6.2% [7], while the United Kingdom observes a prevalence of 7.5% [8]. In Europe, approximately 8,000 hospital-associated infections occur daily, with dozens per hospital each day [7]. In the United States, nearly one in 31 patients experience HAIs during hospital care [9]. The prevalence of HAIs also varies among hospital types [10]. Specifically, prevalence rates for general hospitals, children’s hospitals, maternal and child health hospitals, and oncology hospitals are 3.02% (95% CI, 2.79%-3.26%), 4.43% (95% CI, 3.39%-5.47%), 1.88% (95% CI, 1.47%-2.29%), and 3.96% (95% CI, 3.12%-4.79%), respectively. Furthermore, within a single country, the prevalence may differ across regions. For example, in Beijing, China, the prevalence is 3.6% [11], whereas, in Guangdong province, it is lower at 1.24% [12].

HAIs surveillance is essential for implementing preventive measures, selecting appropriate antibiotic treatments, and reducing infection rates. Objectives include reducing patient mortality and economic burden, identifying risk factors, guiding control practices, and meeting supervision requirements from authorities like the National Centers for Disease Control. Different countries have their own infection-related clinical guidelines. For instance, the National Healthcare Safety Network (NHSN) in the United States published the Patient Safety Component Manual [13], providing classification and diagnostic criteria for hospital-associated infections. In China, the Nosocomial Infection Diagnostic Criteria [14] were issued in 2001 and have not been updated in twenty years. This outdated criteria, which does not cover certain infections like ventilator-associated pneumonia (VAP), neonatal septicemia, and sepsis, may lead clinicians to rely more on personal experience than guidelines. Consequently, patients may receive different infection diagnoses due to the lack of standardized surveillance.

### Information technology for HAIs surveillance

The initial surveillance systems for HAIs are based heavily on manual review of patient records, which are then verified by trained professionals in infection control [15]. However, these approaches are notably time-consuming, and it will take several days or even weeks to manually confirm and complete the infection reports of a tertiary hospital with several thousand beds in China. Automated surveillance systems greatly reduce the workload of hospital infection experts, allowing them to focus on more important aspects of hospital infection prevention. Automated HAIs surveillance systems [16] can be categorized into two types: semiautomated surveillance systems, which aim to assist manual surveillance efforts, and fully automated surveillance systems, which aim to substitute them entirely.

A multitude of studies have demonstrated the efficacy of automated surveillance systems [15, 17] for healthcare-associated infections (HAIs) in reducing the incidence of such infections, as well as the workload associated with manual surveillance and the risks associated with hospital management. In particular, active surveillance of HAIs has been shown to reduce the incidence of bloodstream infections by 44%, urinary tract infections by 28%, and surgical site infections by 20% [18]. Furthermore, a study published in the American Journal of Infection Control in 2018 found that the implementation of an automated HAI surveillance system was significantly associated with a reduction in the incidence of Clostridium difficile infections [19]. The crux of a HAIs surveillance system is the development of an algorithm that can extract the probability of a patient’s healthcare-associated infection from electronic medical record data. This algorithm can be implemented using various approaches, including machine learning [20–22], case-based reasoning, and rule-based methods [23–25].

- Machine learning: The proposed method employs machine learning algorithms (support vector machines (SVM) and gradient tree boosting [26], XgBoost [27], etc.) to classify and predict patient data, with the intention of detecting the presence of infection. Besides traditional machine learning methods, deep neural networks are also used in HAIs surveillance, such as feed-forward artificial neural network (ANN) [28], and long short-term memory neural network (LSTM) [29] The advantages of this method include its ability to automatically learn the features and patterns of infection, but it requires a large amount of data for training and has poor interpretability of the underlying algorithm.
- Case-Based Reasoning (CBR): This method is a paradigm of problem-solving that utilizes past experiences to solve new problems. The primary merit of CBR lies in its ability to manage intricate and indeterminate problems that lack explicit rules. CBR harnesses the knowledge and expertise encapsulated within cases to provide solutions that are contextually pertinent and flexible. Nonetheless, it is worth noting that CBR can incur substantial computational costs, particularly when confronted with extensive case repositories. In addition, its efficacy in reasoning is highly dependent on the accessibility of pertinent and precise cases. In the context of hospital infection monitoring, CBR can be used to identify and analyze similar cases of infections and provide recommendations for treatment and prevention. For example, a CRB system [2] underwent evaluation utilizing real-world data in a Spanish hospital, which produced results indicating that the system reached an accuracy rate of 70.21.
- Rule-based reasoning (RBS): The method relies on an expert system and a rule base to analyze patient data by matching rules in the rule base to determine the presence of infection. The advantage of this method is its ease of understanding and implementation, but it requires experts to develop the rule base. The work of [30] proposes a knowledge-based system that could automatically extract patient data and output possible injury based on knowledge rules on common central line-associated bloodstream infection. The article [31] discusses the development of a rule-based classification system for healthcare-associated bloodstream infections (HABSI) to improve patient safety and infection control. The system achieved high accuracy and correlation coefficients in classifying HABSI according to predefined criteria.

**Fig. 1:**
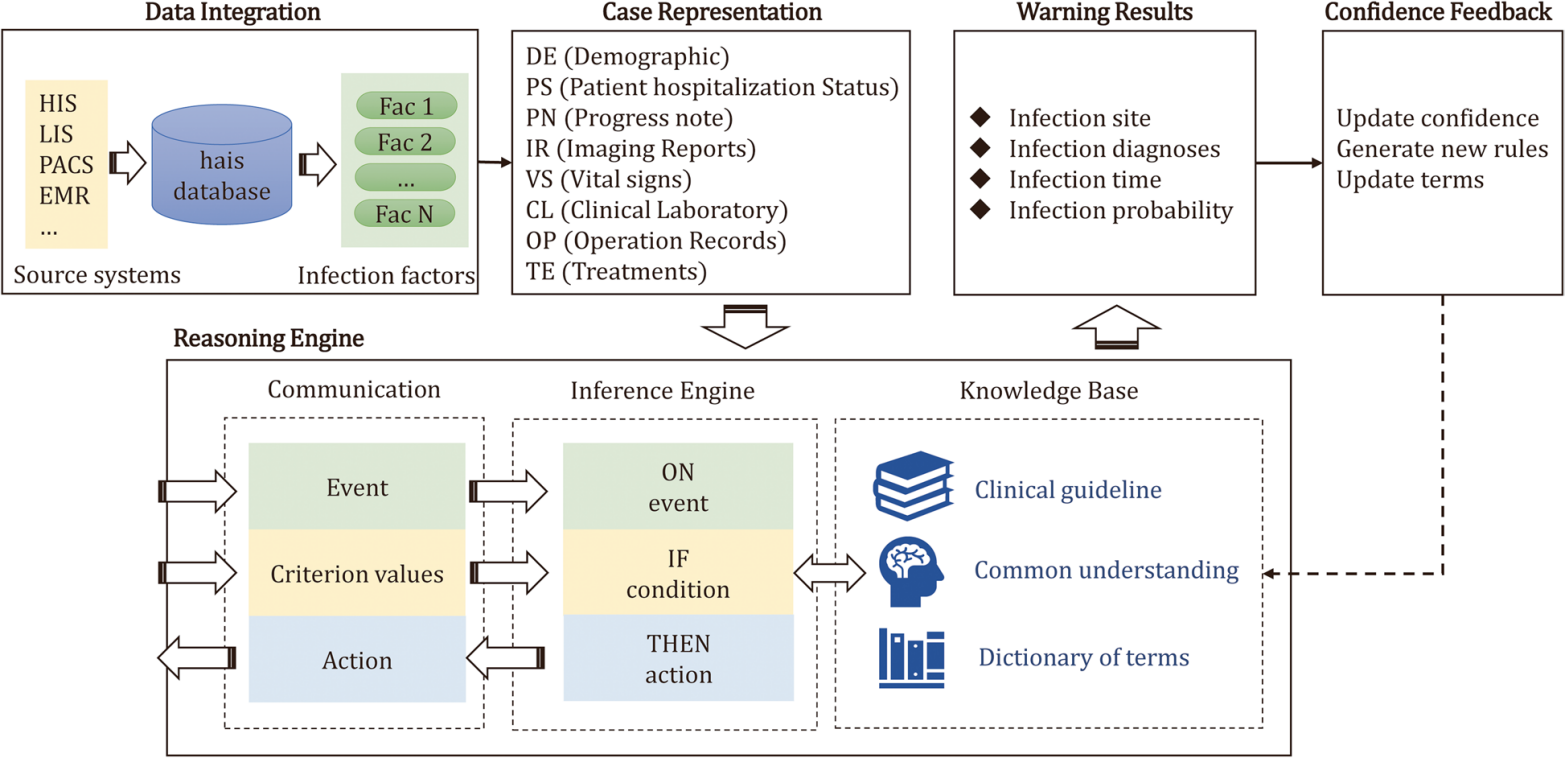
Architecture of the KBHAIS

In addition to its application in HAIs surveillance, the aforementioned methods can be further utilized to assist clinicians in diagnosing infected patients and selecting optimal treatment plans, thus serving as a Clinical Decision Support System (CDSS) [32–36]. In practical implementation, it is critical to get high-quality patient electronic data which plays an equally important role as an appropriate information technology architecture for effective surveillance of HAIs.

So far, the development of surveillance systems for healthcare-associated infections in China [37, 38] is still in its early stages, with a focus on singular or limited types of HAIs or specific departments within hospitals. Work [39] centers on intensive care unit surveillance. Another study [40] meticulously analyzed inpatient data of HAIs cases to identify factors strongly associated with HAIs. Using the decision tree algorithm, a highly accurate and reliable surveillance model was constructed. Similarly, the study of [41] developed an HAIs surveillance system based on the diagnostic criteria for hospital infections (trial implementation) and the diagnostic experience of manual review of medical records. However, the warning conditions associated with this system are relatively simple and cannot provide detailed warning results. We develop a knowledge-based system for the surveillance of healthcare-associated infections, drawing on the knowledge gained from previous research and the practical application of clinical guidelines.

## Methods

In this section, we initially present the comprehensive architecture of our system, elucidating the functionalities of each module. Subsequently, we expound on the method employed to configure the knowledge rules utilized in the system. Following this, we outline the evaluation criteria used to assess the system’s performance. Lastly, we provide detailed information about the dataset used in our study.

### Architecture

As illustrated in Fig. 1, our KBHAIS comprises five distinct modules: *Data Integration*, *Case Representation*, *Reasoning Engine*, *Warning Results*, and *Confidence Feedback*. The Data Integration module focuses on the seamless integration of structured and unstructured data obtained from the healthcare system. Structured data include vital signs such as body temperature, PCT number, CRP number, etc., while unstructured data include progress notes, imaging diagnostic reports, and other relevant information. Before storage, these integrated data undergo a thorough verification and cleansing process. The Case Representation module is responsible for transforming the extracted data into an 8-tuple format, which is subsequently used by the Reasoning Engine module for further processing. The Reasoning Engine module performs a comprehensive analysis of the transformed data. The output generated by the Reasoning Engine module provides detailed infection results, facilitating physicians in their surveillance of HAIs. The Warning Results module plays a crucial role in presenting the findings of the Reasoning Engine module in a concise and informative manner. It provides physicians with clear and actionable warnings, enabling them to take appropriate measures to mitigate the risks associated with HAIs. Moreover, the confidence feedback module ensures dynamic updating of the knowledge base within the Reasoning Engine module. This is achieved through the incorporation of clinician feedback, as well as updates to guidelines and common understandings in the field of HAIs. Details about each module are as follows:

#### Data Integration

A single patient visit generates a vast amount of data that is dispersed across electronic health systems, comprising structured and unstructured data that may contain latent factors associated with the risk of HAIs. To extract these factors from electronic systems, such as the Hospital Information System (HIS), Laboratory Information Management System (LIS), Picture Archiving and Communication System (PACS), and Electronic Medical Record (EMR), the data module focuses on standard interfaces that access these data through database views or web services.

**Fig. 2:**
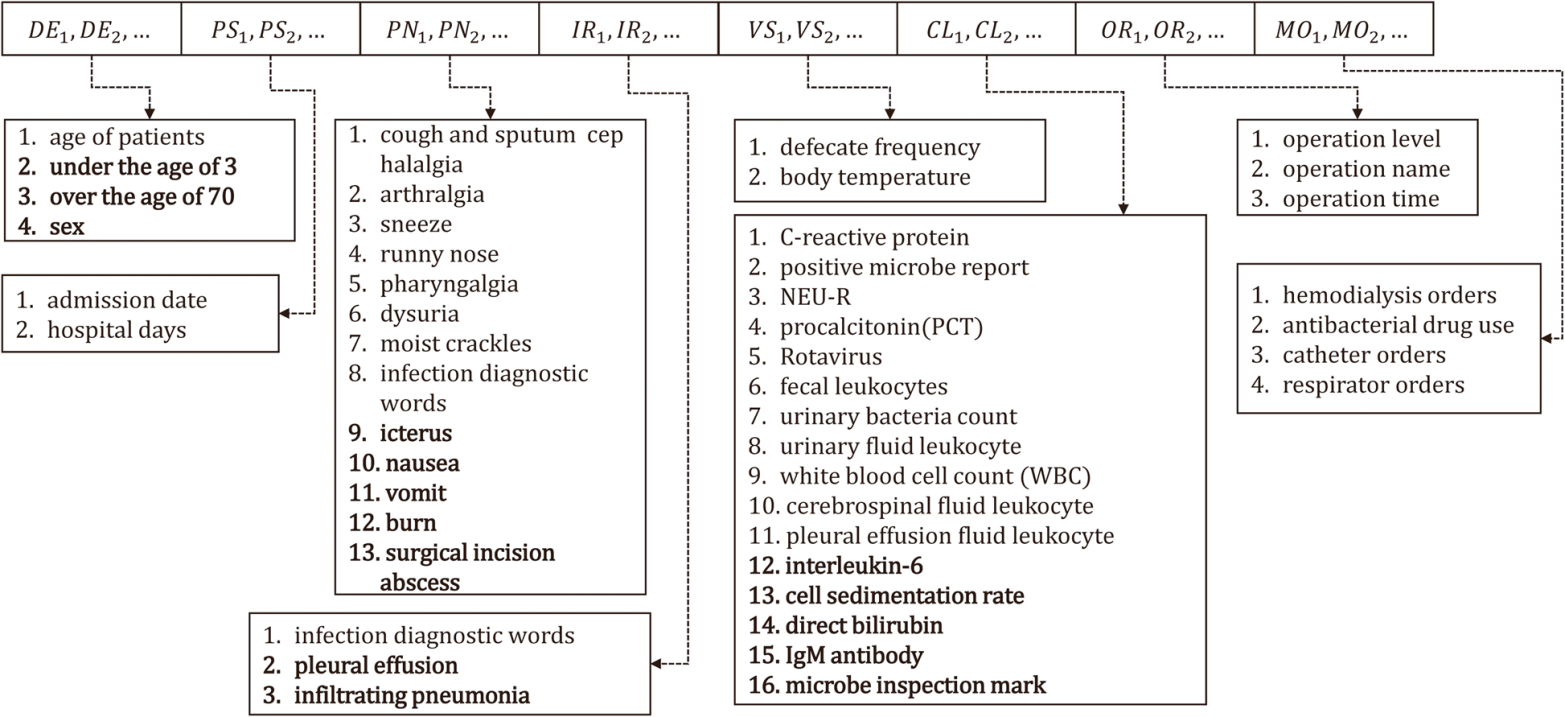
Case representation corresponding to each hospital record sample. Infection factors marked with bold are selected from expert knowledge rules, while others are selected from clinical guidelines

After cleaning and standardization, the original data from various systems are stored in a MySQL database. From these standardized data, the corresponding infection factors can be extracted. For those infection factors that have corresponding standardized code values in the corresponding table structure, such as patient age, gender, and admission time, the extraction process is relatively straightforward. However, for some infection factors, extraction requires processing from continuous numerical values, such as the highest temperature of the day from the temperature chart. In addition, some infection factors need to be extracted from unstructured text, such as information related to patient signs and infection diagnosis in medical records. We have configured the required keywords in our knowledge base to extract corresponding infection factors for each diagnosis.

**Table 1:** List of infection diagnoses for each infection site

Infection site	Infection diagnoses
RTI	Upper respiratory tract infection (UR); Lower respiratory tract infection (LRI); Pleural cavity infection (PCI); Ventilator-associated pneumonia (VAP);
CVS	Endocarditis (ENDO); Myocarditis or pericarditis (CARD);
BSI	Vascular associated infection (VAI); Septicemia(SEP); Transfusion-related infection (TRN-BSI);
AD/GI	Infectious Diarrhea (ID); Gastrointestinal tract infection (GIT); Antimicrobial drug-related diarrhea (ADT); viral hepatitis (VHS); Intraabdominal infection, not specified elsewhere (IAB);Peritoneal fluid infection(PFIT);
CNS	Meningitis or ventriculitis (MEN); Intracranial infection (IC); Spinal abscess/infection (SA);
USI	Non-Catheter-Associated Urinary Tract Infection (UTI); Catheter-Associated Urinary Tract Infection (CAUTI);
SSI	Superficial incisional SSI (SI-SSI); Deep incisional SSI (DI-SSI); Organ/Space SSI (OS-SSI);
BJ	Joint or bursa infection (JNT); Osteomyelitis (OST); Disc space infection (DSI);
SST	Skin infection (SKIN); Soft tissue infection (ST); Decubitus ulcer infection (DECU); Burn infection (BURN); Breast infection or mastitis (BRST); Omphalitis (UMB); Infant pustulosis (IP);
REPR	Episiotomy infection (EPIS); Vaginal cuff infection (VCUF); Pelvic infection (PLI); Endometritis (EMET); Other infections of reproductive tract (OTH-REPR);
ORAL	Oral cavity infection (ORAL)
OTH	Other infections could not be confirmed (OTH)

**Fig. 3:**
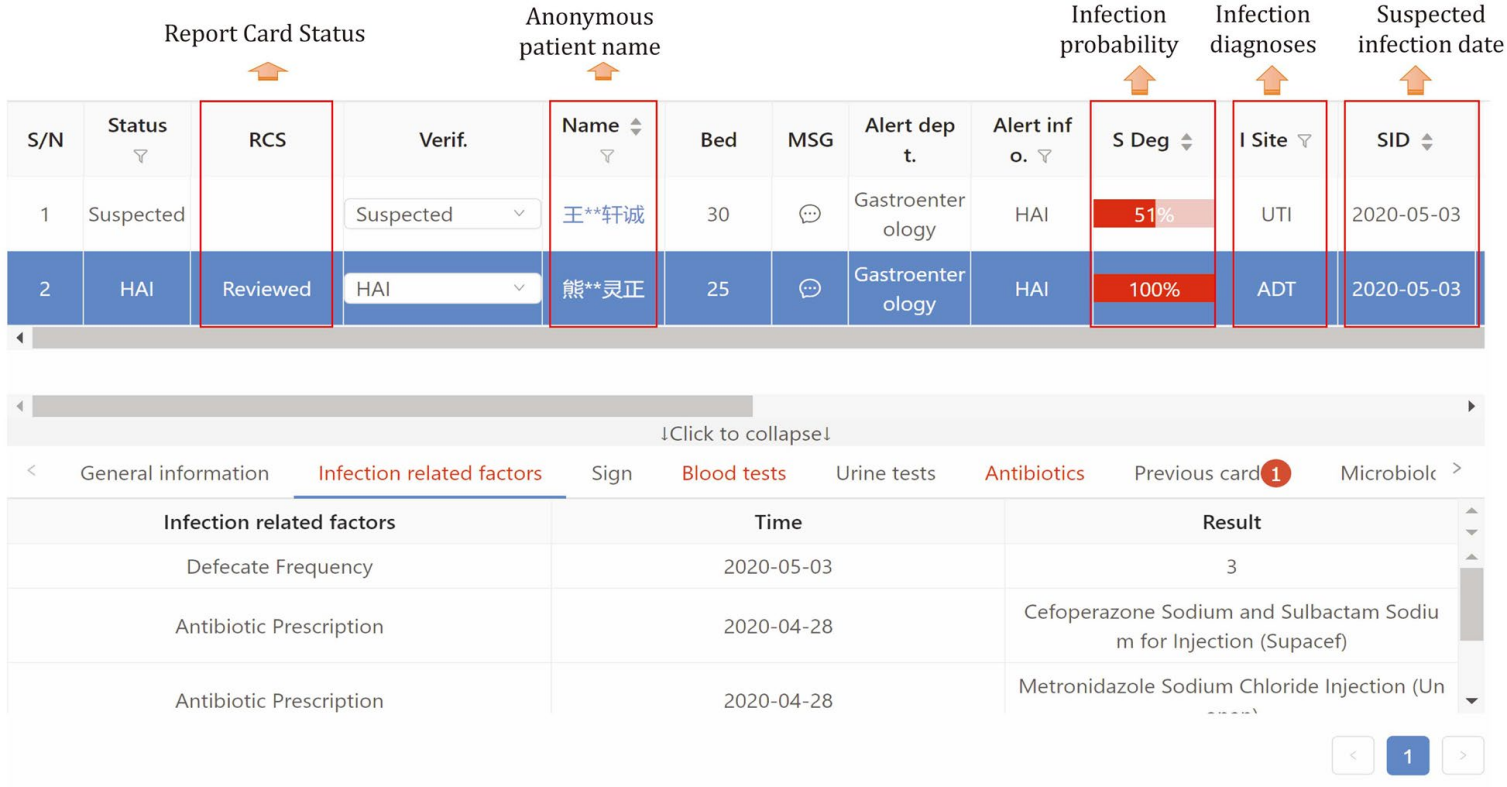
One example of warning result from the KBHAIS

#### Case representation

To enable the system to comprehend the information related to HAIs, it is necessary to transform the data for each patient into a format that the system can interpret. Each patient’s infection-related factors are represented as an 8-tuple DE, PS, PN, IR, VS, CL, OP, MO (Fig. 2) where DE represents demographic characteristics, PS represents patient hospitalization status (e.g., hospital days), PN represents features extracted from progress notes (such as the presence of icterus or a runny nose), IR represents whether diagnostic words about the sites of infection exist in imaging diagnostic reports, VS represents vital signs (for example, body temperature, frequency of defecation), CL represents clinical laboratory-related features, OP represents operation-related features, and MO represents features extracted from procedural medical orders.

There exist two methods to define the values in the Case Presentation tuple. The first approach involves directly extracting infection factors from the Data Integration component. These factors include integer, float, and standardized formatted date types, such as body temperature (float), number of bowel movements (integer), white blood cell count (float), and admission date (date). The second method uses binary type to indicate whether the corresponding infection factor is suspected (0 for unsuspected, 1 for suspected), for example, whether there are diagnostic keywords related to the specific diagnosis in the medical progress notes and whether there are positive bacterial cultures.

#### Reasoning engine

The reasoning engine consists of three components, including *Communication*, *Interface Engine*, and *Knowledge Base*. The function of each component is described below:

- Communication: In conjunction with the interface engine, the communication mechanism manages the prepared case representation and the warning results. Specifically, if a case exhibits critical factors associated with infection, the communication mechanism transmits the case to the inference engine component. Subsequently, if the rule interface engine yields results indicating that the patient has an infection, the communication mechanism will alert the user. In contrast, if the results indicate no likelihood of infection, the communication mechanism will not take any action. Notably, if the results indicate that the patient has hospital-associated infections (HAIs) within a repeat infection timeframe (RTI), a period used to prevent the same infection site from recurring within a predetermined time window, the communication component will not take action. The RTI value is set to fourteen days in accordance with clinical guidelines.
- Interface Engine: The inference engine is mainly responsible for processing the knowledge rules, using prepared, structured information generated from the case representation engine to execute the logic. The interface engine determines whether these rules should be implemented based on the patient’s infection factors. When all conditions in one knowledge rule are satisfied, this rule is triggered, and the corresponding output is produced based on the action configurations. In practice, we use drools [42] to manage and process knowledge rules. Drools is an open-source Business Engine that is based on the RETE algorithm. In drools, knowledge rules could be implemented as DRL files using LHS language and decision tables. It is easy to access enterprise policies, easy to adjust, and easy to manage.
- Knowledge rule base: The knowledge base plays a vital role as the brains of our system for identifying infections. The crucial point of this component is that each knowledge rule is stored in a way that machines can understand and execute by the inference engine. We extract knowledge rules from clinical infection guidelines and infection experts’ domain knowledge The latter refers to the shared knowledge and experience of infection preventionists, while clinical guidelines are based on a rigorous review of the available scientific literature. In China, the clinical guidelines for hospital infection prevention and control are updated relatively slowly and may not fully cover the actual needs of HAIs prevention and control. Therefore, in practical HAIs surveillance work, the knowledge of infection preventionists is extremely valuable. We consulted relevant preventionists and translated their experience into knowledge rules to supplement the infection monitoring knowledge rule library.

In general, the reasoning engine infers data from inpatients to determine whether they have a healthcare-associated infection which follows the explanation criteria in the Nosocomial Infection Diagnostic Criteria [14], including:

- Infections with no clear incubation period, which occur within 48 hours of admission, are considered hospital infections. Infections with a clear incubation period are considered hospital infections if they occur after the average incubation period has passed since admission.
- Infections directly related to a previous hospitalization.
- Infections caused by potential infections activated by diagnostic and treatment measures, such as herpes virus and tuberculosis.
- Infections acquired by newborns during delivery or postpartum.

#### Warning results

Unlike previous work with only probabilities of infection, our system gives a detailed warning result which contains the infection time, the infection sites, the type of infection and the possibility of infection (Fig. 3). A clinical physician must confirm every detailed warning result. Confirmed healthcare-associated infection cases are stored in the system and reported to China Center for Disease Control and Prevention. Besides, clinical physicians should manually record infection cases that are not warned by our system. Knowledge rules are added or modified according to confirmed warning lists.

#### Confidence feedback

Each rule possesses a confidence level, which reflects the probability of infection. During the training phase, the confidence level *Pr*(*i*) is initialized based on expert experiences and iteratively updated on a monthly basis using Eq. 1.

1 $$\begin{aligned} Pr\left( i\right) =Pr\left( i-1\right) +\alpha \frac{Acc\left( i\right) -Pr\left( i\right) }{Pr\left( i-1\right) } \end{aligned}$$

where $Pr\left( i-1\right)$ represents the confidence of one certain rule for the last iteration, *Acc*(*i*) represents the accuracy of the rule for this iteration, $\alpha$ as the adjusting parameter, and we set $\alpha =0.75$ for practice. As a result, the higher the accuracy of a specific rule, the higher the probability of the corresponding warning case.

Since physicians in the hospital infection department need to check each warning case one by one and report patients diagnosed with hospital infections, we need to maintain a high level of sensitivity while also ensuring specificity, and it is possible that a greater number of warnings may be generated than anticipated by clinical physicians. To strike a balance between the accuracy of the warning and the workload of the physicians, we implement a configurable threshold mechanism. Each infection diagnosis has a corresponding threshold configured. If the probability of infection of an alert falls below this threshold, it will not be sent to the infection department doctors. The configurable threshold can be adjusted according to actual work needs in practical applications.

**Fig. 4:**
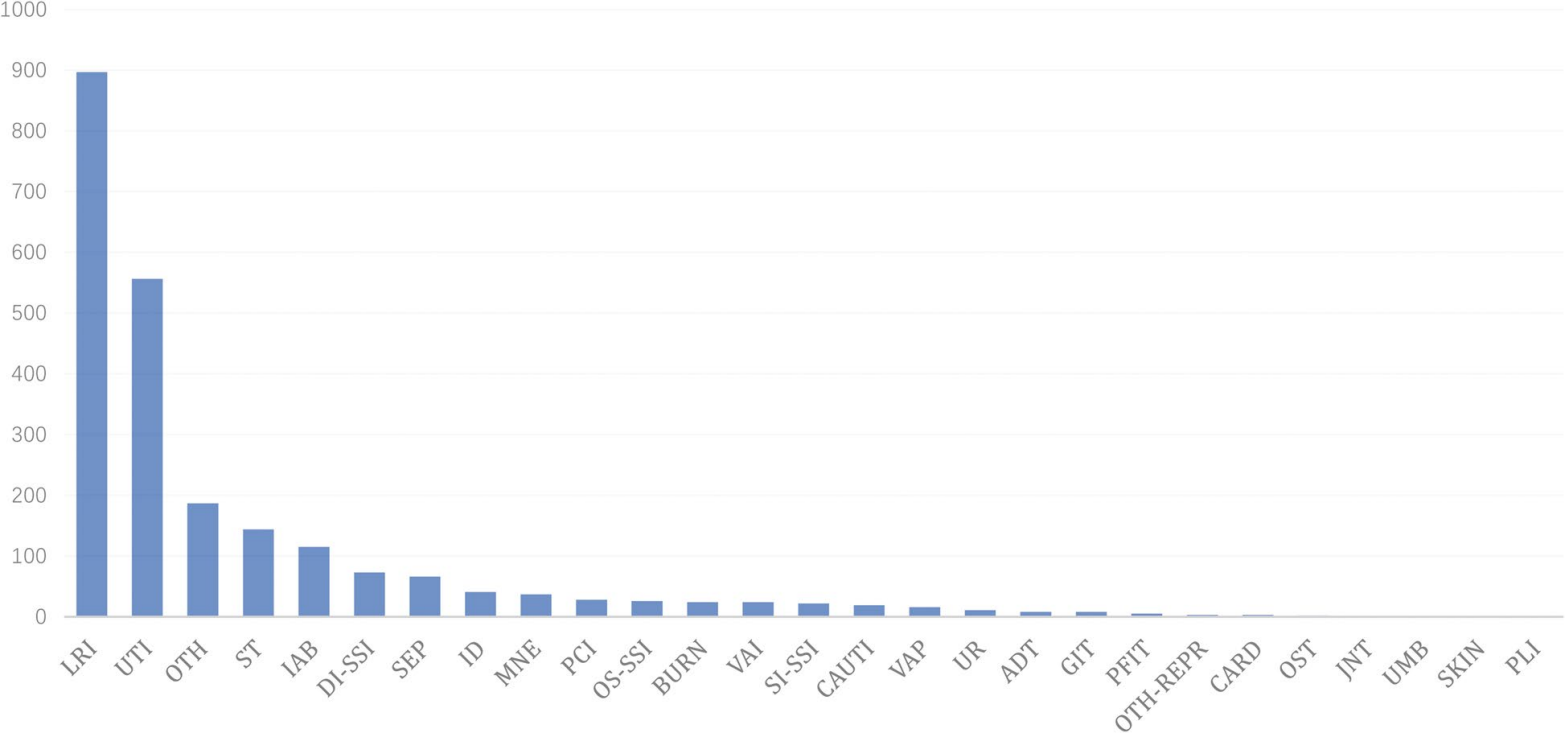
Distribution of different HAIs types in Gansu Provincial Hospital

**Fig. 5:**
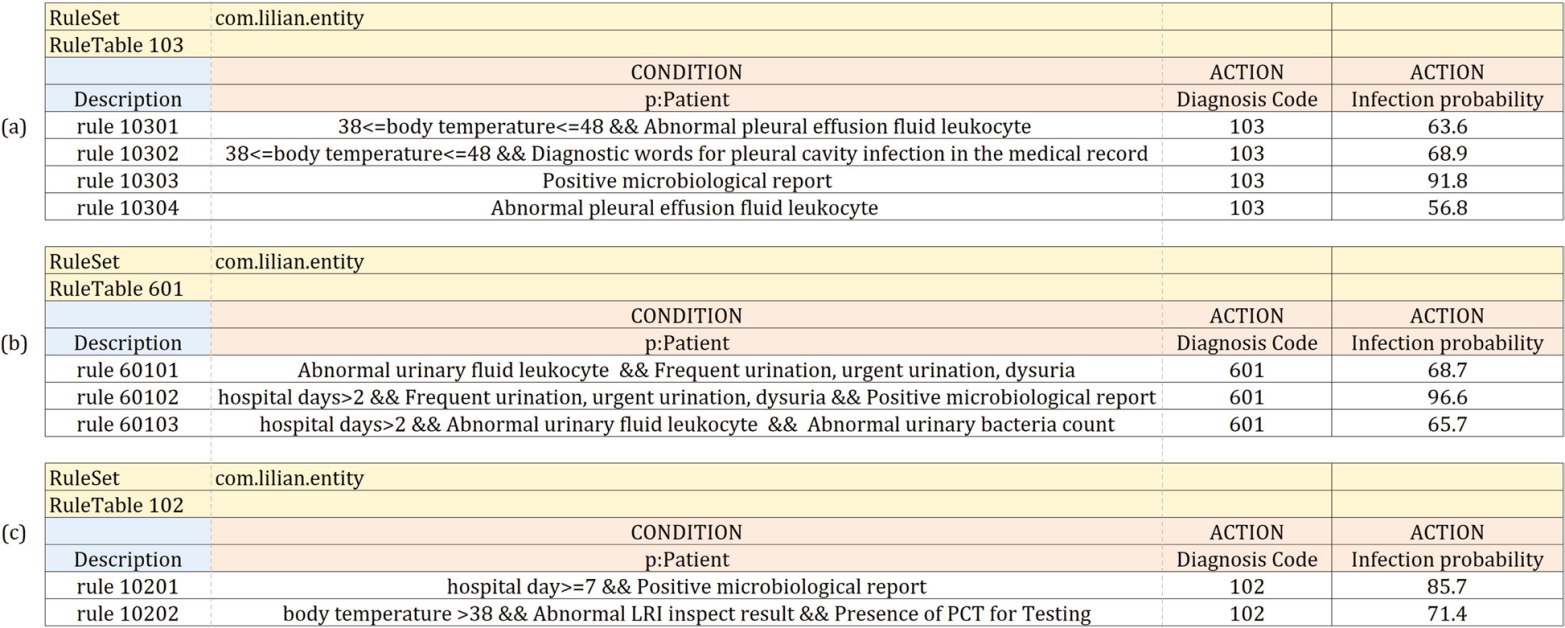
Example of a simplified version of the decision tables: **a** Knowledge rules for pleural cavity infection according to clinical guidelines. **b** Knowledge rules for urinary system infection according to clinical guidelines. **c** Knowledge rules for lower respiratory tract infection according to the common understanding of experts

### Configuration

The KBHAIS underwent two stages of optimization and validation. In the optimization stage, knowledge rules based on clinical guidelines are initialized with a certain degree of confidence. New expert knowledge rules and infection-related terms are subsequently added to the knowledge base under expert supervision. In the validation stage, the knowledge rules, rule confidence, and infection-related terms remain unchanged and consistent with the end of the training stage. The clinical infection guidelines used for extracting knowledge rules include:

- Nosocomial Infection Diagnostic Criteria, which is issued by the National Health Commission of the People’s Republic of China
- Guidelines for the diagnosis and treatment of Community-Acquired Pneumonia (CAP) in Chinese adults
- Expert consensus on the diagnosis and treatment of neonatal septicemia 2018
- Guidelines for sepsis In China 2018

We classify healthcare-associated infections into 40 distinct types that are categorized into 12 infection sites, as illustrated in Table 1. Our classification is based on a thorough analysis of four clinical guidelines and expert opinions. We have extracted a total of 154 knowledge rules from the clinical guidelines and 52 from experts’ common understanding. The knowledge rules with initialized confidence for all types of infections can be found in the supplementary material. We have provided three examples of the knowledge rules that we have identified below.

- Pleural Cavity Infection: Based on the pleural cavity infection criteria outlined in the Diagnostic Criteria for Healthcare-Associated Infections in China, the presence of chest pain, purulent or foul-smelling pleural effusion, and a white blood cell count greater than $1000 \times 106/L$ in routine examination suggest the possibility of pleural cavity infection. Based on clinical diagnosis, a definitive diagnosis can be made if bacteria are observed in pleural effusion or smear. It can be inferred that the diagnosis of pleural cavity infection depends on five key elements: body temperature range, diagnostic words in the progress note, diagnostic words in the imaging diagnostic report, pleural effusion fluid leukocyte, and microbiological inspection report. These elements are combined into different knowledge rules in the form of a drools decision table, as shown in Fig. 5(a). The term “Ruleset" refers to the worksheet being a ruleset, “com.lilian.entity" refers to the corresponding Java packages, and “p:Patient" refers to the corresponding Java class. Each white line represents a complete rule. When all the “CONDITIONS" in the same line are satisfied, “ACTIONS" will be triggered to record one infection event with the detailed rule ID (e.g., “10301") and the infection probability. If more than one rule is satisfied for one infection diagnosis simultaneously, only the rule with the highest infection probability will be triggered and executed.
- Urinary System Infection: The diagnostic criteria for urinary system infection consist of clinical and etiological criteria. These criteria are divided into two categories: UTI (Non-Catheter-Associated Urinary Tract Infection) and CAUTI (Catheter-Associated Urinary Tract Infection), depending on whether or not the patients are associated with catheters. According to the urinary system infection clinical guidelines, seven infection elements are identified for UTI, which include body temperature range, hospital days, urinary fluid leukocyte number, urinary fluid leukocyte abnormal flag, urinary bacteria count abnormal flag, records about frequent urination, urgent urination and dysuria in the progress note, and microbiological inspection report. As shown in Fig. 5(b), a drools decision table summarizes the knowledge rules based on these elements. The element "Hospital days" differentiates between hospital-acquired and community-acquired infections. Hospital-acquired infection is defined as an infection that occurs within 48 hours after admission, as there is no definite incubation period.
- Lower respiratory tract infection: Clinical physicians use some infection factors not included in clinical guidelines. These factors include hospital day, patient age, and whether the patient has a lab of PCT, among others. A drools decision table for lower respiratory tract infection is presented in Fig. 5(c). As it is not always possible to accurately obtain every infectious factor involved in clinical guidelines in the system, the experience and common understanding of experts can serve as a good supplement to the rule base for improving the sensitivity of warnings for hospital-acquired infections (HAIs). To this end, we have compiled 52 rules from infection experts.

**Table 2:** HAIs surveillance performances of different months in Gansu Provincial Hospital

	2020												2021		
	Jan	Feb	Mar	Apr	May	Jun	Jul	Aug	Sep	Oct	Nov	Dec	Jan	Feb	Mar
SEN $\spadesuit$	0.828	0.763	0.833	0.773	0.752	0.815	0.786	0.725	0.771	0.773	0.830	0.609	0.782	0.603	0.843
SEN $\spadesuit$+$\clubsuit$	**0.849**	**0.763**	**0.860**	**0.801**	**0.789**	**0.838**	**0.799**	**0.759**	**0.836**	**0.822**	**0.887**	**0.674**	**0.813**	**0.667**	**0.862**
SPE $\spadesuit$	**0.881**	**0.868**	**0.824**	**0.827**	**0.842**	**0.840**	**0.840**	**0.984**	**0.979**	**0.979**	**0.971**	**0.978**	**0.877**	**0.867**	**0.853**
SPE $\spadesuit$+$\clubsuit$	0.880	0.867	0.823	0.827	0.841	0.839	0.839	0.982	0.976	0.976	0.968	0.975	0.875	0.867	0.852
ACC $\spadesuit$	**0.881**	0.866	**0.825**	**0.828**	**0.841**	**0.841**	**0.840**	**0.981**	**0.977**	**0.976**	**0.970**	**0.976**	**0.877**	**0.866**	**0.853**
ACC $\spadesuit$+$\clubsuit$	0.880	**0.867**	0.824	0.828	0.841	0.840	0.840	0.980	0.975	0.975	0.968	0.974	0.876	0.866	0.853

### Evaluation

Our system is a semi-automated hospital infection monitoring system, which means that all infection alerts must be confirmed and reviewed by Infection Preventionists (IPs) before generating an infection report and uploading it to the CDC National Center. In China, sensitivity is the most concerned criterion because the Chinese Center for Disease Control and Prevention surveillance requires that each healthcare-associated infection case be reported. This means we must include infected patients in our warning results as much as possible. We choose the performance measures [17] accuracy (ACC), sensitivity (SEN), and specificity (SPE) to evaluate our system. These measures are defined as Eq. 2:

2 $$\begin{aligned} \text {ACC}{} & {} = \frac{\left( TP + TN\right) }{\left( P+N\right) } \nonumber \\ \text {SEN}{} & {} = \frac{TP}{\left( TP + FN\right) } \nonumber \\ \text {SPE}{} & {} = \frac{TN}{\left( TN + FP\right) } \end{aligned}$$

where True Positive (TP) refers to cases where the system correctly identifies a patient with a healthcare-associated infection as having an infection. Conversely, False Positive (FP) occurs when the system incorrectly flags a patient as having an HAI, even though they do not have an infection. False Negative (FN) refers to cases where the system fails to detect a patient with an actual HAI, resulting in a failure to raise an alert. True Negative (TN) includes cases where the system correctly identifies a patient without an HAI as not having an infection.

The definition of patients with infections is based on authentic infection reporting card records. In practical clinical settings, hospital infection reporting cards are initially completed by clinical physicians (the patient’s attending doctors) and subsequently reviewed and verified by IPs. When using our system, the patient’s attending physician daily reviews the warning information generated by our system. If they confirm a patient with a hospital-acquired infection, they report it. In contrast, if the warning is erroneous, it is excluded. Additionally, if the patient’s attending physician identifies a patient not listed among the hospital-acquired infection patients alerted by our system, they can manually include the case in the reporting. Ultimately, all reporting card information undergoes a thorough review and verification process by IPs.

We conducted a monthly evaluation of accuracy (ACC), sensitivity (SEN), and specificity (SPE) from January 2020 to March 2021. Furthermore, we performed an assessment of sensitivity (SEN) for different infection diagnoses and sites during the two stages. To compare the performance of different configurations of knowledge rules, we design two scenarios ($\spadesuit$) and ($\spadesuit$+$\clubsuit$). The first scenario represents the configuration with knowledge rules based only on clinical guidelines. In contrast, the second scenario represents configuration with knowledge rules based on clinical guidelines and the common understanding of experts simultaneously.

### Dataset

Surveillance of HAIs through the analysis of real-world electronic medical data is a complex endeavor. To tackle this challenge, we partnered with Gansu Provincial Hospital, a renowned grade A tertiary care hospital in China with extensive clinical expertise in infection prevention and control. Our study utilized a dataset of 106,769 samples collected from 84,839 inpatients, both with and without HAIs, from January 2020 to March 2021. The data was extracted from four electronic medical systems, including HIS, LIS, PACS, and EMR. Our analysis initially focused on the distribution of infection types, with practical infection cases obtained from statistics reported to the Chinese Center for Disease Control and Prevention. As demonstrated in Fig. 4, the types of infection exhibit a long-tail distribution. Our findings reveal that lower respiratory tract infection (LRI) is the most commonly observed infection, followed by non-catheter-associated urinary tract infection (UTI) and soft tissue infection (ST), which is consistent with preventionists opinion.

The initial phase is dedicated to the development and testing of the system, while the subsequent phase involves its implementation and validation. The data collected is divided into two distinct sets based on the admission date. Specifically, patients admitted between January and December 2020 are utilized for the first stage, whereas those admitted between January and March 2021 are utilized for the second stage.

## Results

### Effectiveness of different stage

Table 2 presents the sensitivity, specificity, and accuracy metrics for both stages. As expected, the results indicate that the incorporation of expert knowledge to supplement the knowledge of clinical guidelines has led to a certain degree of improvement in sensitivity. In particular, the sensitivity ranges from 75% to 90% in most months, with the exception of February 2021 and December 2020. On the other hand, specificity and accuracy metrics outperform sensitivity. This is attributed to the fact that the number of patients with healthcare-associated infections is a small fraction of the overall inpatient population, as previously stated. However, compared to the configuration with knowledge rules based solely on the common understanding of experts, specificity and accuracy metrics have decreased slightly with the configuration that incorporates knowledge rules based on infection-related clinical guidelines and the common understanding of experts simultaneously.

### Effectiveness of infection types and infection sites

Regarding the system’s capability to identify a specific type of infection, our warning mechanism encompasses a comprehensive range of forty distinct infection diagnoses. The findings of the top 10 sensitivity for infection diagnoses are presented in Table 3. The results indicate that in the training stage, infectious diarrhea (ID) exhibits the highest sensitivity, while in the testing stage, deep incisional surgical site infection (DI-SSI) demonstrates the best sensitivity. Furthermore, DI-SSI and vascular-associated infection (VAI) also exhibit excellent sensitivity at the training stage, with a value exceeding 90%. In contrast, catheter-associated urinary tract infection (CAUTI) shows the lowest sensitivity at 56.5 percent.

Our KBHAIS system not only has the ability to accurately classify each type of healthcare-associated infection, but it can also correctly identify the site of infection. Since each infection diagnosis is associated with a specific site, the precision and sensitivity performance for the infection type are similar to those for infection diagnoses. In Table 4, the sensitivity of different infection sites is presented for both stages. The results indicate that the bloodstream infection event (BSI) and the abdomen / gastrointestinal system infection (AD / GI) exhibit the best sensitivity of greater than 90% for both scenarios. In addition, the infection diagnosis of infectious diarrhea (ID) in Table 4 has the best sensitivity and is associated with the AD/GI site. This finding is consistent with the notion that better sensitivity to infection diagnosis typically leads to better sensitivity to identify the site of infection.

**Table 3:** Sensitivity performances for infection diagnoses among the top 10 sensitivity results in the HAIs surveillance

Stage	Diagnoses	ID	DI-SSI	VAI	VAP	LRI	IC	ST	IAB	UTI	CAUTI	**AVG**
2020	SEN $\spadesuit$	0.960	0.944	0.915	0.881	0.871	0.860	0.756	0.750	0.632	**0.565**	0.813
	SEN $\spadesuit$+$\clubsuit$	**0.980**	**0.944**	**0.931**	**0.976**	**0.878**	**0.940**	**0.785**	**0.875**	**0.889**	0.492	**0.849**
2021	SEN $\spadesuit$	0.958	0.968	0.917	0.900	0.880	0.833	0.696	0.650	0.679	**0.625**	0.814
	SEN $\spadesuit$+$\clubsuit$	**0.958**	**0.980**	**0.964**	**0.900**	**0.899**	**0.833**	**0.768**	**0.850**	**0.875**	0.581	**0.863**

**Table 4:** Sensitivity performances for different infection sites in the HAIs surveillance

Stage	Infection site	RTI	CVS	BSI	AD/GI	CNS	USI	SSI	SST	BJ	REPR	ORAL	AVG
2020	SEN $\spadesuit$	0.872	0.735	0.906	0.901	0.896	0.582	0.836	0.687	0.667	0.727	0.600	0.764
	SEN $\spadesuit$+$\clubsuit$	**0.878**	**0.816**	**0.921**	**0.926**	**0.955**	**0.644**	**0.896**	**0.712**	**0.857**	**0.818**	**0.800**	**0.838**
2021	SEN $\spadesuit$	0.878	0.455	0.908	0.820	0.800	0.645	0.833	0.672	0.400	0.600	0.500	0.683
	SEN $\spadesuit$+$\clubsuit$	**0.896**	**0.818**	**0.954**	**0.885**	**0.867**	**0.709**	**0.917**	**0.738**	**0.667**	**0.800**	**0.956**	**0.841**

Tables 5 and 6 demonstrate the system’s exceptional performance across various diagnoses and sites, with minimal variation. This can be attributed to the fact that true positive cases constitute only a small proportion of the overall dataset when evaluating specific infection diagnoses and sites. As a result, the system tends to yield a higher proportion of True Negative (TN) cases, leading to elevated specificity values. However, it is noteworthy that Lower Respiratory Infections (LRIs), which comprise the largest proportion of actual infections, exhibit relatively lower specificity compared to other diagnoses. LRIs belong to the category of Respiratory Tract Infections (RTIs), where the specificity is comparatively lower than other infection sites.

**Table 5:** Specificity performances for infection diagnoses among the top 10 sensitivity results in the HAIs surveillance

Stage	Diagnoses	ID	DI-SSI	VAI	VAP	LRI	IC	ST	IAB	UTI	CAUTI	AVG
2020	SPE $\spadesuit$	0.998	0.997	0.997	0.996	0.974	0.999	0.984	0.996	0.997	0.997	0.994
	SPE $\spadesuit$+$\clubsuit$	0.998	0.998	0.997	0.997	0.974	0.999	0.984	0.996	0.994	0.997	0.993
2021	SPE $\spadesuit$	0.998	0.996	0.997	0.999	0.954	0.999	0.971	0.994	0.995	0.995	0.990
	SPE $\spadesuit$+$\clubsuit$	0.998	0.998	0.997	0.999	0.954	0.999	0.971	0.994	0.993	0.997	0.990

**Table 6:** Specificity performances for different infection sites in the HAIs surveillance

Stage	Infection site	RTI	CVS	BSI	AD/GI	CNS	USI	SSI	SST	BJ	REPR	ORAL	AVG
2020	SPE $\spadesuit$	0.933	0.998	0.994	0.984	0.997	0.971	0.993	0.938	0.985	0.997	0.999	**0.981**
	SPE $\spadesuit$+$\clubsuit$	0.933	0.998	0.994	0.984	0.997	0.968	0.993	0.938	0.985	0.997	0.999	0.981
2021	SPE $\spadesuit$	0.883	0.997	0.993	0.970	0.997	0.956	0.993	0.868	0.967	0.995	0.999	**0.965**
	SPE $\spadesuit$+$\clubsuit$	0.884	0.997	0.993	0.971	0.997	0.955	0.993	0.869	0.967	0.995	0.999	0.965

## Discussion

In this study, we develop a knowledge-based surveillance system for the purpose of healthcare-associated infection surveillance. Our approach has demonstrated good sensitivity, and the sensitivities obtained by our system have shown promising results when comparing different infection sites and diagnoses. With hundreds of inpatients and dozens of episodes of hospital-acquired infections that occur each day, it is highly labor-intensive and time-consuming for physicians of the Department of Hospital Infection Management to filter out all infected patients from thousands of inpatients. Our system dramatically reduces the workload of surveillance and screening of hospital-acquired patients. It should be noted that the actual sensitivity of our approach is higher than the statistical results indicate. This is due to our system being designed to avoid alerting physicians again if they have already identified an infected patient in advance of the alert consequence. This feature helps to reduce confusion and workload for physicians.

In addition to knowledge rules extracted from infection-related clinical guidelines, knowledge rules extracted from the common understanding of experts could also improve the sensitivity of the system. This demonstrates the high value of our system for replication. If we update the knowledge base to include more expert experience and extract more knowledge rules from other healthcare providers, it will result in a precious infection diagnosis brain.

Table 2 shows that performances in February 2021 and December 2020 are not as satisfactory as in other months. We speculate that this is because, during these two months, physicians are more proactive in identifying infected patients in advance of system alerts. During the last month of the year and the month of Chinese New Year, physicians are usually extremely busy, which can lead to a lag in the writing of medical records, resulting in a lag in the acquisition of infection factors associated with medical records, thus reducing the sensitivity of early warnings.

Table 3 suggests that the sensitivity of our system in detecting urinary system infections and catheter-associated urinary tract infections is inadequate when utilizing knowledge rules extracted solely from clinical guidelines. The factor "urine pain" within the UTI rules is presented in Fig. 5(b) is subjective data that does not have a corresponding diagnostic code within the system. These subjective factors may not be easily extracted from our EMR systems, which rely on doctors to record them accurately and timely in medical records. The absence or delay of these records can affect the effectiveness of early warning. To address this issue, we can extract more objective factors from clinical guidelines, such as pathogen test results, or use expert knowledge rules that cover alternative infection factors not included in clinical guidelines. This approach can reduce the impact of difficult-to-extract objective factors on the warning effectiveness. As anticipated, the addition of knowledge rules extracted from the common understanding of experts can effectively increase the sensitivity of UTI warnings. However, it has no significant effect on CAUTI. Further optimization and enhancement of knowledge rules regarding CAUTI are necessary.

Table 4 shows that our KBHAIS has a satisfactory sensitivity for each infection site. Still, the knowledge rules extracted from the common understanding of experts vary in the degree to which the sensitivity is improved. When both types of knowledge rules are used, USI, BJ, and ORAL performances significantly improve compared to a configuration with knowledge rules extracted from infection-related clinical guidelines only. This indicates that the clinical guidelines for USI, BJ, and ORAL infections have lagged somewhat relative to expert experience and need to be updated and supplemented.

Compared with previous work, our system has numerous advantages, including:

- Knowledge-based approach: Our system is based on the knowledge rules extracted from the infection guidelines and the common understanding of experts. To the best of our knowledge, this approach is unique in China and has not been implemented before in the surveillance of healthcare-associated infections.
- Comprehensive coverage: Our system provides warnings for forty different HAIs belonging to twelve different infection sites, significantly improving over previous systems that only provided warnings for one or a few types of infections.
- Detailed warning results: Compared to previous work, the warning results of our system are more detailed, specifically including the site of infection, time of infection, probability of infection, and type of infection.
- High accuracy: Evaluation results show that our system has satisfactory sensitivity and specificity. This high level of accuracy helps clinicians to reduce the rate of delayed and missed reporting of healthcare-associated infections.

Overall, our system significantly improves over previous work in healthcare-associated infection surveillance. Its knowledge-based approach, comprehensive coverage, detailed warning results, and high accuracy make it a valuable tool for clinicians in China.

## Conclusion

We develop a knowledge-based system to help clinicians in the surveillance and prevention of healthcare-associated infections. The system is well designed and capable of (1) automatically extracting infection factors from patient electronic health records, structured and unstructured, (2) integrating knowledge rules derived from clinical experts and clinical guidelines, and (3) ensuring that the decision-making process is transparent and traceable. Specifically, we have extracted 154 knowledge rules from clinical guidelines issued by relevant authorities and an additional 52 knowledge rules from the shared expertise of clinical experts. These knowledge rules are activated when the corresponding infection factors are detected. The system generates results that include infection diagnosis, probability of infection, infection type, and infection site, which are then sent to infection preventionists for review.

We hereby present the validation of our knowledge-based surveillance system for healthcare-associated infections conducted at Gansu Provincial Hospital. Specifically, we conducted a comparative analysis between the results obtained from knowledge rules derived solely from guidelines and those derived from both guidelines and the collective expertise of domain experts. The findings reveal that the system exhibited an average sensitivity exceeding 83%, which underlines its notable effectiveness.

The importance of our research for future work is mainly reflected in the following aspects: (1) The system can automatically extract infection factors from electronic health records and integrate knowledge rules from clinical experts and clinical guidelines. This provides a valuable resource for researchers to discover and validate new knowledge related to infections. Through the analysis of large-scale medical data, researchers can explore the complex relationships among infection factors, identify novel infection risk factors, and further optimize the system’s knowledge base. (2) The system ensures transparency and traceability in the decision-making process, which is crucial for guiding surveillance practice and formulating healthcare policies. Researchers can utilize the system’s decision process data to evaluate the effectiveness and rationality of medical decisions, providing evidence and reference for future practices and healthcare policymaking. (3) By integrating knowledge rules from clinical experts and guidelines, the system can provide efficient and accurate preventive measures. Researchers can analyze the system’s output to assess the effectiveness of existing infection prevention and treatment strategies and offer improvement suggestions to enhance infection control measures.

Looking ahead, several areas for improvement have been identified. First, system stability and practicability require further verification using a larger dataset from multiple hospitals in future work. Second, while our system demonstrates strong sensitivity to most infections, improvements are needed for urinary system infections and catheter-associated urinary tract infections. This challenge could be addressed by incorporating expert knowledge from multiple medical facilities. Third, given the poor precision observed for intraabdominal infection and soft tissue infection, efforts should be made to minimize the number of warnings while maintaining sensitivity. Finally, we plan to integrate more advanced deep learning technologies to enhance the extraction of accurate factors from unstructured data from electronic medical records.

### Supplementary Information

**Additional file 1.**

## Data Availability

The datasets generated and/or analyzed during the current study are not publicly available due to the confidential requirements of Gansu Provincial Hospital. Still, anonymous sample data is available from the corresponding author Haojun Zhang (haozi_523@163.com) on reasonable request.
